# The effect of a randomised controlled lifestyle intervention on weight loss and plasma proneurotensin

**DOI:** 10.1186/s12902-022-01183-4

**Published:** 2022-10-31

**Authors:** Louise Bennet, Ayesha Fawad, Joachim Struck, Sara Lönn Larsson, Andreas Bergmann, Olle Melander

**Affiliations:** 1 Department of Clinical Sciences, Lund University, Malmö, Sweden; 2 Clinical Trial Centre, Clinical Studies Sweden – Forum South, Skåne University Hospital in Lund, Jan Waldenströms gata 35, 205 02 Malmö, Sweden; 3 Lund University Diabetes Center, Lund University, Malmö, Sweden; 4 Sphingotec GmbH, Hennigsdorf, Germany; 5 Waltraut Bergmann Foundation, Hohen Neuendorf, Germany; 6 Department of Internal Medicine, Skåne University Hospital, Malmö, Sweden; 7 Metabolic Center, Region Skåne, Malmö, Sweden

**Keywords:** Proneurotensin, Culturally adapted lifestyle intervention, Weight loss, Middle East, Type 2 diabetes, Migration

## Abstract

**Aims:**

Proneurotensin (Pro-NT) is a strong predictor of cardiometabolic disease including type 2 diabetes and obesity, however, the effect of lifestyle change on Pro-NT has not been investigated in this context. Middle Eastern (ME) immigrants represent the largest and fastest growing minority population in Europe and are a high-risk population for obesity and type 2 diabetes. In this randomised controlled lifestyle intervention (RCT) addressing ME immigrants to Sweden where weight-loss was previously studied as the main outcome, as a secondary analysis we aimed to study change in Pro-NT during follow-up and if baseline Pro-NT predicted weight loss.

**Methods:**

Immigrants from the Middle East at high risk for type 2 diabetes were invited to participate in this RCT adapted lifestyle intervention of four months’ duration. The intervention group (N = 48) received a culturally adapted lifestyle intervention comprising seven group sessions and a cooking class addressing healthier diet and increased physical activity. The control group (N = 44) received treatment as usual with information to improve lifestyle habits on their own. Data assessed using mixed effects regression.

**Outcomes:**

Primary outcome; change in Pro-NT. Secondary outcome; change in BMI in relation to baseline plasma concentration of Pro-NT.

**Results:**

During the four months follow up, weight was significantly reduced in the intervention (-2.5 kg) compared to the control group (0.8 kg) (β -0.12, 95% CI -0.24 to -0.01, *P* = 0.028). Pro-NT increased to a significantly greater extent in the intervention compared to the control group during follow up (28.2 vs. 3.5 pmol/L) (β 11.4; 4.8 to 18.02, *P* < 0.001). Change over time in BMI was associated with baseline Pro-NT (β 0.02; 0.01 to 0.04, *P* = 0.041).

**Conclusion:**

In consistence with data from surgical weight loss, this RCT paradoxically shows increased levels of Pro-NT during a multifactorial lifestyle intervention resulting in weight loss. Long term studies of Pro-NT following weight loss are needed.

**Trial registration:**

This study is a secondary analysis of the RCT trial registered at www.clinicaltrials.gov. Registration number: NCT01420198. Date of registration 19/08/2011. The performance and results of this trial conform to the CONSORT 2010 guidelines.

## Introduction

Type 2 diabetes represents a growing health concern contributing to increased morbidity in diabetic macro- and microvascular complications but also to impaired quality of life, high societal and health-related costs and with increased mortality rates corresponding to shorter life courses. Type 2 diabetes is increasing globally and the World Health Organization estimated that in 2014 over 420 million people had type 2 diabetes, which corresponded to a global prevalence of 8.5% ^1^. Some areas in the world, such as the Middle East, reach endemic levels with up to 20–30% of the population affected [1].

Type 2 diabetes is a consequence of either pure insulin deficiency or relative insulin deficiency caused by long-lasting insulin resistance [2]. Our previous data showed that the Middle Eastern immigrant population to Sweden is, to a higher degree, compared to the native Swedish population, insulin resistant. One can argue that insulin resistance is due to obesity and unhealthy lifestyle habits that cluster in this population, however, irrespective of the influence of those contributing risk factors, the insulin resistance is still more profound in this population as compared to the native Swedish population thus indicating presence of an altered fat metabolism [3].

Neurotensin is a gut hormone that is involved in intestinal fat absorption [4], but is also produced and active within the central nervous system where it is shown to regulate satiety [5–8]. It consists of a 13-amino acid peptide, and binds to three different receptors, neurotensin receptor 1 (Ntsr 1), 2 (Ntsr2) and 3 (Ntsr3) (15, 16). Circulating neurotensin primarily comes from the gut and is stimulated by fat intake [8]. Importantly, since neurotensin is unstable both invitro and invivo, it is difficult to accurately measure. By contrast, the stable 117-amino acid fragment from the N-terminal part of the pre-Pro-NT/neuromedin precursor hormone referred to as Pro-NT, which is released in a 1:1 ratio with the mature hormone, can be easily and accurately measured [9].

High circulating concentration of Pro-NT is associated with risk of obesity, type 2 diabetes, breast cancer and with total and cardiovascular mortality [10].

Pro-NT is a strong predictor of cardiometabolic disease, however, the effect of lifestyle change on Pro-NT has not been investigated. In order to increase the understanding of contributing mechanisms to altered fat metabolism and obesity in this high risk population for obesity and type 2 diabetes, the aim of this randomized controlled trial (RCT was (1) to investigate the impact of lifestyle change on Pro-NT levels and (2) to investigate if baseline levels of Pro-NT predicted weight reduction during the intervention. This was a secondary analysis in a previous randomised controlled lifestyle intervention addressing weight-loss and cardiometabolic change as the main outcome.

## Methods

### Study population

The randomised controlled lifestyle intervention, which was conducted in 2015 in the city of Malmö, Sweden, concerned immigrants from the Middle East at high risk for developing type 2 diabetes [11]. The aim of the intervention was to improve insulin sensitivity, weight reduction and reduce type 2 diabetes risk [12]. To summarise, individuals identified in the previous MEDIM population based study (the impact of Migration and Ethnicity on Diabetes In Malmö, conducted 2010 to 2012) born in the Middle East (Iraq) and that were at risk for type 2 diabetes by being overweight (> 28 kg/m^2^) were invited to participate (N = 636) [11]. in the study. A total of 104 individuals wanted to participate in the study and were invited for a baseline health examination. At that examination, eight individuals were identified with type 2 diabetes and were excluded from the study. No participants reported history of other chronic diseases including cardiovascular disease, cancer, hypertension or chronic obstructive lung disease. Participants were randomised to intervention or control with a 1:1 allocation ratio using a random number generator as previously described in detail [11]. The planned intervention was initially of three years’ duration and was meant to include both Iraqi born and native Swedes. However, due to shortage of resources, the intervention only included Iraqi born participants with a follow-up time reduced to four months duration. The lifestyle intervention was culturally adapted where the intervention group received counselling in their native language (Arabic) on healthy habits including identification of cultural and social barriers for lifestyle change together with motivational and economic support for lifestyle change as previously described in detail [11]. The intervention group also participated in cooking classes, supervised by a professional chef with lifelong experience cooking food for diabetes patients [13]. Participants were encouraged to bring their favourite recipe, that was modified to include lower total fat as well as saturated fat percentage, and higher proportion of fibres [13]. The control group received ‘treatment as usual’, i.e., a counselling consisting of 30 min health information provided by an Arabic and Swedish speaking nurse, ordinarily working with diabetes patients and with long experience of providing information of healthy lifestyles. The health information was provided orally and in writing where the nurse informed what lifestyle habits to improve and suggestions how to improve these habits.

**Fig. 1:**
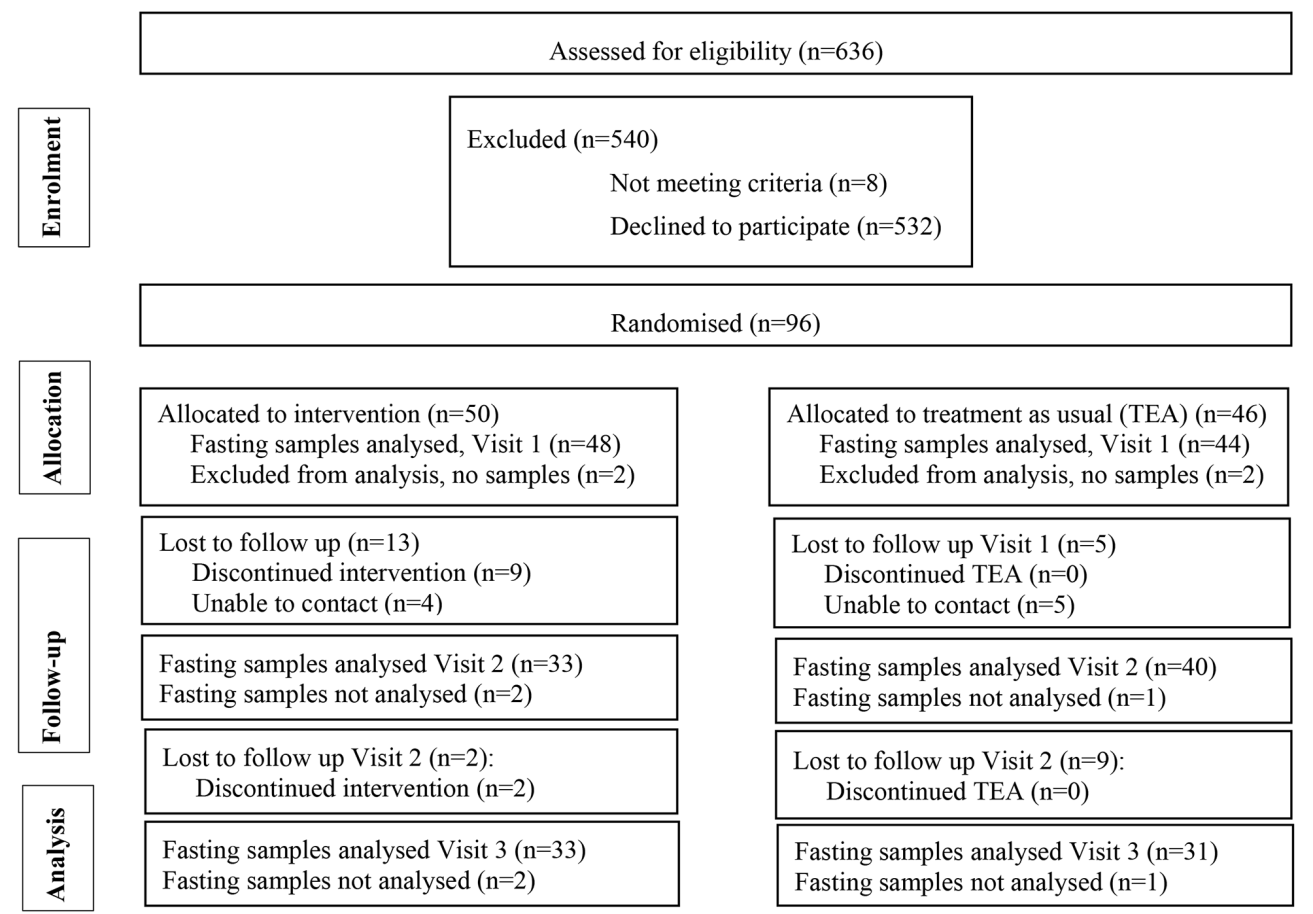
Flow diagram of the progress through the phases of the RCT including enrolment, intervention allocation, follow-up, and data analysis. “Visit 1” Baseline, “Visit 2” two months follow up, “Visit 3” four-months follow up

As mentioned, this study was a secondary analysis in a previous randomised controlled lifestyle intervention addressing weight-loss and cardiometabolic change as the main outcome. The goals for the lifestyle intervention were based on the Finnish diabetes prevention study and Diabetes Prevention Program aiming to reach 5% reduction in body weight, achievement of 30 min physical activity per day, increased fibre intake and reduced total fat and saturated fat intake as detailed previously [11]. The study was conducted from mid-January 2015 to mid- June the same year, i.e., four-months’ duration. This study was registered on 19/08/2011, at www.clinicaltrials.govNCT01420198. The Consort Statement was applied [12]. A flow chart describing enrolment, intervention allocation, follow-up and data analysis is presented in Fig. 1.

Measurement of Pro-NT from baseline and at least one more time point was required for the participant to be included in the final assessments.

### Assessments

Fasting samples, oral glucose tolerance tests and questionnaires capturing self-reported lifestyle habits, comorbidity and medication were assessed at baseline, 2- months and at 4 months (end of study) [11]. Self-reported physical activity was assessed using International Physical Activity Questionnaire (IPAQ) and self-reported diet intake was assessed using 4-day diet diaries. In addition, physical activity was objectively assessed using Actigraph® GT3X. Assessment of standard physical examinations, fasting samples, OGTT and clinical variables such as blood pressure, height, weight, body mass index (BMI) and waist-circumference was performed as described previously [11].

Pro-NT was measured using a chemiluminescence based immunoassay as described in detail previously (sphingotec GmbH, Hennigsdorf/Berlin, Germany) [9].

### Outcome

Primary outcome was change over time in Pro-NT and secondary outcome was change over time in body mass index in relation to baseline plasma concentration of Pro-NT.

### Statistical analysis

Statistical analysis was assessed using IBM SPSS version 24.0, STATA (version 13) and R Studio version 3.6.0. Differences between groups at baseline were assessed using an independent sample t-test for normally distributed continuous data and Mann-Whitney U test for non-normally distributed continuous data. Categorical variables were compared using chi-square test or Fisher’s exact test .

Restricted maximum likelihood estimation of mixed effects regression was used to estimate the parameters in the models and to assess the effect of the intervention on the outcomes with respective change in Pro-NT and BMI respectively between the intervention group and the control group. The correlation within-subject errors due to the repeated measurements were adjusted by adding random intercepts and random slopes in addition to the fixed effects. Advantage with this model is that it uses all available observations during the study period and no imputation of missing data was needed. The difference in slopes of the outcomes as function of time between the intervention and the control groups was estimated by the time by group interaction (time * group) variable. The coefficient estimates are expressed in original units together with standardized regression coefficients. We developed this model is three steps. Firstly, a crude model was developed including only group, follow-up time and the group × time interaction. Secondly, we adjusted for age, sex, and education. Thirdly, we also added baseline value of fasting glucose and insulin sensitivity index (ISI).

As a sensitivity analysis we repeated the analysis on the subset of participants with post-baseline observations, corresponding to a per-protocol set analysis.

Level of significance was set to ≤ 0.05 and no multiple testing was done.

To the best of our knowledge, this is the first RCT on Pro-NT and lifestyle change in at-risk individuals of Middle Eastern background. Therefore, we did not have any estimates in change of Pro-NT. Thus the power calculation in this RCT was assessed rather on change in fasting glucose levels from baseline to end of intervention [14]. Further, due to the low participation rate, we used restricted likelihood estimation of mixed regression; thus the initial power calculation that required a larger sample size was no longer relevant for this study.

## Results

### Baseline characteristics

At baseline there were no differences between the intervention (N = 48) and control groups (N = 44) regarding age and sex distribution, body weight, blood pressure or biomarkers for cardiovascular disease including Pro-NT, lipids, glucose (fasting and 2 h) or HbA1c (Table 1). A higher level of education was more prevalent in the control than in the intervention group otherwise there were no differences in socioeconomic situation or lifestyle habits assessed as physical activity and food intake between the intervention and control groups. Non-participants were younger and had higher BMI and lower physical activity levels than participants.

**Table 1:** Baseline characteristics in the MEDIM intervention study

Variables	Intervention n = 48	Control n = 44	*P*-value
Age (years)	47.9 (10.4)	48.9 (9.05)	0.63
Male sex, n (%)	23 (46)	22 (47.8)	1.00
Proneurotensin, pmol/L, median (IQR^+++^)	119.3 (92.8–156.0)	104.5 (90.4–156.6)	0.69
Body weight (kg)	85.5 (15.2)	80.0 (13.1)	0.06
Body mass index(kg/m^2^)	31.0 (4.4)	29.6 (3.6)	0.09
Waist circumference men(cm)	108.2 (9.6)	106.1 (8.9)	0.45
Waist circumference women(cm)	100.7 (8.1)	97.2 (8.1)	0.13
Insulin Sensitivity Index, median (IQR^+++^)	62.4 (46.0, 99.5)	73.3 (56.7, 118.0)	0.16
Systolic blood pressure (mmHg)	121.6 (13.3)	126.9 (16.1)	0.08
Diastolic blood pressure (mm Hg)	77.6 (8.4)	79.7 (11.2)	0.30
Fasting glucose (mmol/L)	5.6 (0.5)	5.4 (0.7)	0.25
2-h glucose (mmol/L)	6.6 (1.8)	6.5 (1.8)	0.87
HbA1_C_ (mmol/mol) HbA1_C_ in %	34 (4.7) 5.3% (0.4)	35(4.4) 5.4% (0.4)	0.33
Lipid-lowering drugs, n (%)	1 (2)	0 (0)	1.00^∞^
LDL-cholesterol (mmol/L)	3.4 (0.7)	3.3 (0.9)	0.51
HDL-cholesterol (mmol/L)	1.3 (0.3)	1.3 (0.4)	0.97
First-degree Family history T2D, n (%)	34 (68)	24 (52.2)	0.14
Education < HS^+^, n (%)	7 (14)	16 (34.8)	**0.03**
Unemployment, n (%)	26 (52)	24 (52.2)	1.00
Smoking, n (%)	8 (16)	8 (17.4)	0.86
Physical activity (MET^++^-hours/week), median (IQR^+++^)	8.5 (0-47.6)	12.4 (0-66.3)	0.32^*^
Moderately active^**^, n (%)	18 (36)	26 (56.5)	0.05
Total fat intake (g/d)	86.1 (29.3)	98.3 (34.6)	0.08
Total carbohydrate intake (g/d)	198.92 (63.4)	187.53 (71.2)	0.06
Total protein intake (g/d)	85.92 (30.8)	74.66 (19.8)	0.05
Mean caloric intake (kcal)	1886.3 (619)	2052.3 (616)	0.21

Data presented as mean (standard deviation), numbers (percentages) or median (interquartile range). Differences between groups were compared using independent sample t-test for continuous variables and chi-square test for categorical variables

^+^High school; ^++^ Metabolic Equivalent of Task; ^+++^Interquartile range; ^*^Mann-Whitney U-test; ^∞^ Fisher’s exact test;

^**^ Individuals accumulating 10 MET-hours/week;

### Change over time in Pro-NT and BMI

**Fig. 2:**
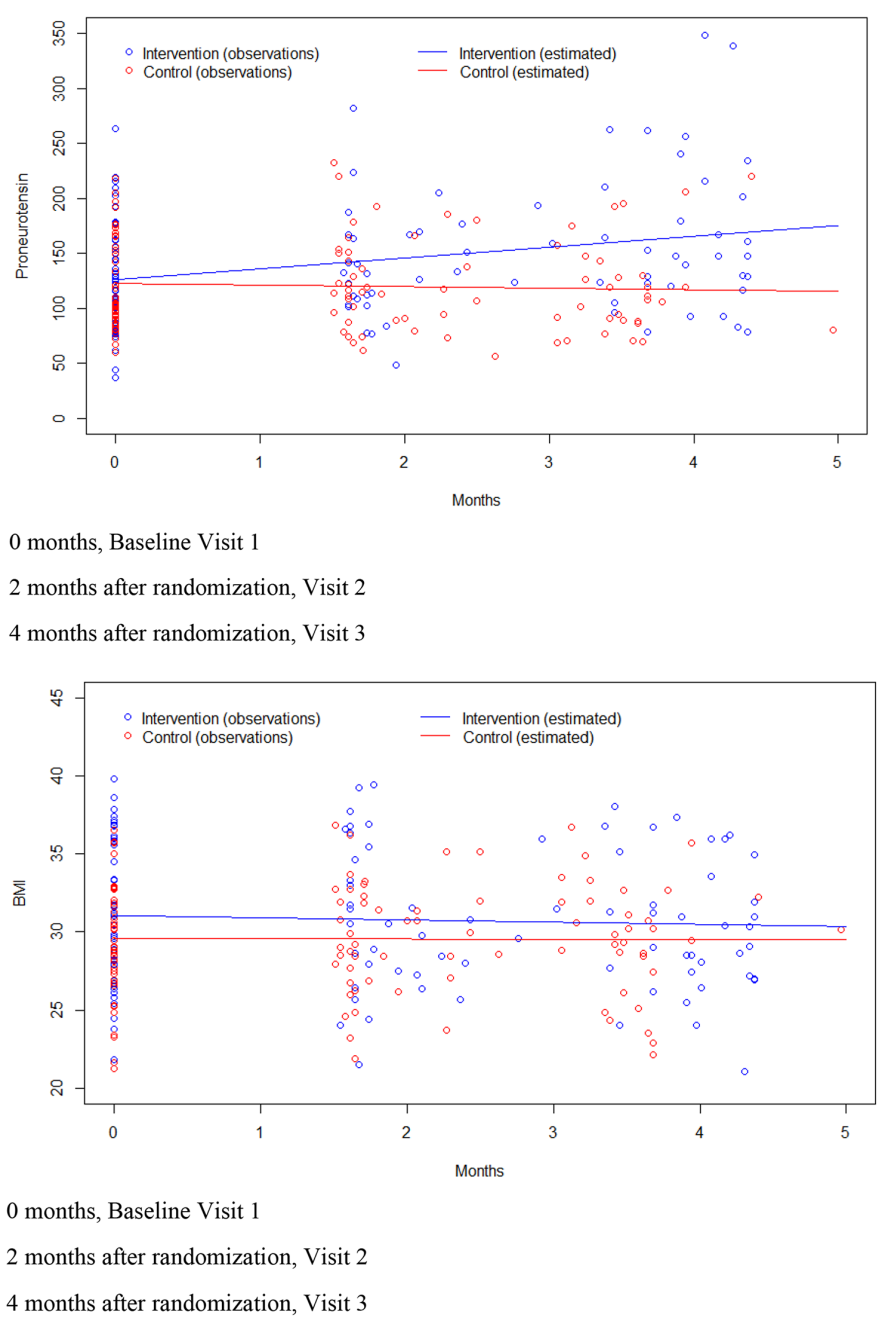
**a**: Linear prediction plot of Pro-NT for four-months follow-up in the intervention and control groups, unadjusted data **b**: Linear prediction plot of body mass index (BMI) for four-months follow-up in the intervention and control groups, unadjusted data

The results are presented for the intention-to-treat (ITT) participants. The drop-out rate was similar in both groups and at the last (third visit) 68.7% (N = 33) and 70.4% (N = 31) participants respectively remained in each group. There was an increase in Pro-NT at the second and third visit compared to the baseline visit in the intervention group but not in the control group (Table 2). Descriptive change over time in Pro-NT is illustrated in Fig. 2a.

**Table 2:** Descriptive changes over time in outcomes at visit 1, visit 2 and visit 3 in the MEDIM intervention study

Variables	Intervention				Control			
	**Visit 1 (n = 48)**	**Visit 2 (n = 33)**		**Visit 3 (n = 33)**		**Visit 1 (n = 44)**	**Visit 2 (n = 40)**	**Visit 3 (n = 31)**
Proneurotensin (pmol/L) (Median, IOR)	119.3 (92.8–156.0)		133.2 (109.6–167.0)	147.5 (118.0–213.0)		104.5 (90.4–156.6)	115.5 (89.5–151.1)	108.0 (88.1–143.2)
Proneurotensin (natural logarithm)	4.78 (0.40)		4.89 (0.35)	5.03 (0.41)		4.75 (0.33)	4.75 (0.35)	4.72 (0.34)
Body weight (kg)	85.5 (15.2)		85.2 (15.6)	83.0 (15.0)		80.0 (13.1)	80.8 (12.8)	80.8 (13.8)
Body mass index (kg/m^2^)	31.0 (4.4)		30.8 (4.5)	30.4 (4.3)		29.6 (3.6)	29.6 (3.6)	29.6 (3.7)
Physical activity (MET^+^-hours/week), median (IQR^++^)	8.5 (0-47.6)		65.8 (20.6-101.2)	62.6 (23.1-151.1)		12.4 (0-66.3)	27.1 (1.9–126)	24.0 (6.3–129)
Total fat intake (g/d)	86.1 (29.3)		75.9 (28.3)	75.2 (25.8)		98.3 (34.6)	96.2 (32.9)	91.1 (30.0)
Mean caloric intake (kcal)	1886.3 (619)		1740.4 (557.6)	1692.6 (553.8)		2052.3 (616)	2055.9 (568)	1941.3 (511.4)

Data presented as mean (standard deviation). ^+^ Metabolic Equivalent of Task ^++^Interquartile range

Visit 1 Baseline

Visit 2 Two months after randomization

Visit 3 Four months after randomization

In a crude analysis, the difference in change of Pro-NT from baseline to follow-up between the intervention and control group was significant, confirmed by an interaction between time and group status (Table 3). With four-months duration, this corresponds to a mean difference in change of Pro-NT of 48.75 pmol/L (95% CI = (20.44, 77.07). Change over time in Pro-NT, was only modestly affected in the adjusted models (Table 3). As a sensitivity analysis, we repeated the analysis on the per-protocol-set (PPS) and the results were in line with results in the ITT data set; model in Table 2 adjusted for age, sex, education and BMI, mean difference in change of Pro-NT of 46.66 pmol/L (19.95, 73.37) during 4 months.

**Table 3:** Mixed effects regression analysis of proneurotensin. Associations are presented as β as well as well as standardized β coefficients with 95% confidence intervals CI’s, studying group*time interaction, adjusting for group status, follow-up time, age, sex, education, baseline BMI, fasting glucose and baseline insulin sensitivity index (ISI).

Variables	β (95% CI)	Standardized β	p-value	β (95% CI)	Standardized β	p-value	β (95% CI)	Standardized β	p-value
Follow-up time, months	-1.41 ( -6.04, 3.20)	-0.60	0.549	-1.57 (-6.19, 3.03)	-0.67	0.505	-2.54 (-7.44, 2.46)	-0.98	0.328
Group status (Intervention group)	3.55 (-13.34, 20.47)	0.41	0.681	0.07 (-17.09, 17.31)	0.01	0.994	-2.47 (-20.41, 15.34)	-0.27	0.787
Group status*time^a^	11.24 (5.02, 17.48)^***^	3.54^***^	< 0.001	11.35 (5.13, 17.57)^***^	3.58^***^	**< 0.001**	11.44 (4.82, 18.02)^***^	3.40^***^	**< 0.001**
Age, years				0.37 (-0.43, 1.18)	0.89	0.372	0.55 (-0.27, 1.37)	1.27	0.205
Male sex				14.75 (-0.84, 30.33)	1.82	0.069	15.51 (-0.60, 31.60)	1.82	0.068
Education (high vs. low)				9.08 (-9.73, 27.87)	0.93	0.354	11.00 (-8.52, 30.53)	1.07	0.287
Baseline BMI kg/m^2^				1.44 (-0.49, 3.37)	1.44	0.151	1.98 (-0.0002, 3.96)	1.90	0.058
Baseline fasting blood glucose mmol/l							-0.76 (-13.94, 12.38)	-0.11	0.912
Baseline ISI							0.07 (-0.04, 0.18)	1.21	0.228

^*^ P < 0.05

^**^ P < 0.01

^***^ P < 0.001

Change in Pro-NT correlated with change in BMI in the intervention (0.48, 0.16–0.71) but not in the control group. We found no correlations neither in the intervention nor in the control group between Pro-NT vs. ISI, Pro-NT vs. fasting glucose or between BMI vs. ISI.

Descriptive change over time in BMI is illustrated in Fig. 2b. There was a significant interaction between group status and time (Table 4) showing that the decrease of BMI was larger in the intervention than in the control group independent of anthropometrics or baseline Pro-NT level. Although change over time in BMI was only modestly dependent on the other included covariates, baseline Pro-NT within the level of significance was associated with change over time in BMI.

The models were also adjusted for physical activity and caloric intake/total fat intake, but these variables were not associated with the outcomes, and not included in the models Tables 3 and 4.

**Table 4:** Mixed effect regression analysis of body mass index (BMI). Associations are presented as β as well as well as standardized β coefficients with 95% confidence intervals CI’s, studying group*time interaction, adjusting for group status, follow-up time age, sex, education, baseline proneurotensin, baseline fasting glucose and baseline insulin sensitivity index

Variables	β (95% CI)	Standardized β	p-value	β (95% CI)	Standardized β	p-value	β (95% CI)	Standardized β	p-value
Follow-up time, months	-1.01 ( -0.09, 0.06)	-0.39	0.696	-0.01 ( -0.09, 0.06)	-0.39	0.699	-0.02 (-0.10,0.07)	-0.36	0.715
Group status (Intervention group)	1.47 (-0.15, 3.10)	1.77	0.076	1.61 (-0.07, 3.28)	1.85	0.064	2.08 (0.34,3.82)	2.264	**0.024**
Group status*time^a^	-0.13 (-0.02, -0.03)^*^	-2.46^*^	**0.014** ^*^	-0.13 (-0.02, -0.03)^*^	-2.45^*^	**0.014** ^*^	-0.12^*^ (-0.24, -0.01)	-2.19^*^	**0.028**
Age, years				-0.01 (-0.09, 0.08)	-0.12	0.902	-0.03 (-0.12, 0.06)	0.68	0.497
Male sex				0.47 (-0.14, 2.08)	0.56	0.574	0.15 (-1.55, 1.84)	0.16	0.871
Education (high vs. low)				0.62 (-2.56, 1.32)	-0.62	0.547	-055 (-2.59, 1.49)	-0.51	0.609
Baseline proNT pmol/L							0.02 (0.01, 0.04)^*^	0.57^*^	**0.041**
Baseline fasting blood glucose mmol/l							0.41 (-0.97, 0.07)	2.05	0.571
Baseline ISI							-0.01 (-0.02, 0.01)	-0.47	0.637

^*^ P < 0.05

^**^ P < 0.01

^***^ P < 0.001

## Discussion

In this four-months RCT study, the intervention subjects clearly displayed increased Pro-NT levels as well as reduced weight during follow-up compared to the control group.

The effect of lifestyle change on Pro-NT has, to the best of our knowledge, not been investigated previously in humans. As previously reported the Iraqi immigrant population in Sweden is at high risk for cardiometabolic disease reflected by the fact they have a twice as high risk for obesity and type 2 diabetes as compared to the native Swedish population [15]. The intention of this study was not to investigate or distinguish between genetically driven aspects of Pro-NT variation and culturally/food driven aspects across Middle Eastern and European ethnicities. Rather, we wanted to investigate the impact of lifestyle change on Pro-NT levels and if baseline levels of Pro-NT predicted changes in BMI during follow up.

Participants’ fat intake in this lifestyle intervention at baseline was generally high (approximately over 40% of total fat intake) [13]. Our data shows that baseline Pro-NT was associated with increased levels of BMI over time which is consistent with previous studies showing that elevated plasma Pro-NT predicts obesity, type 2 diabetes, breast cancer and cardiovascular disease later in life, supporting a pro-obesity net effect by high neurotensin levels [10].

Pro-NT mediates regulation of body weight via different tissues. Peripherally acting Pro-NT promotes fat absorption and weight gain, whereas central Pro-NT signaling suppresses feeding and weight gain. It has been discussed as an anti-obesogenic agent which could explain our paradoxical findings of increased Pro-NT levels with weight loss. For instance, Ratner et al. (2016) have shown that neurotensin (both circulating and peripheral) regulates appetite, decreases food intake and has an anorexigenic effect and, furthermore, that these effects are abolished by a neurotensin antagonist [16]. In both rat models as well as in humans, gastric bypass induced weight loss has, in consistency with our data, shown elevated levels of neurotensin months after surgery [16, 17]. In contradiction, neurotensin-deficient mice are reported to be protected against obesity because of neurotensin-induced decreased intestinal fat absorption [4]. The increased levels of Pro-NT following weight loss could reflect other compensatory mechanisms and defence towards hunger in order to restrain weight gain. One possible explanation to increased Pro-NT levels after surgical weight loss is that Pro-NT production is elevated to compensate for lipid malabsorption induced by bariatric procedures. With post-surgical diets low in fat, elevated plasma Pro-NT may not be sufficient to enhance fat accumulation. A possible side-effect of increased plasma Pro-NT is increased transport of Pro-NT across the blood-brain barrier and augmenting Pro-NT levels in the hypothalamic regions that regulate energy balance [18]. Thus, it is unclear if the satiety effect of high Pro-NT is transient or sustained and consequently if it is beneficial in clinical practice.

Although population based data has previously shown that Pro-NT is more strongly associated with glucose regulation in Middle Easter immigrants than the native Swedish population, implying that neurotensin has a stronger negative influence on glucose regulation and development of diabetes and obesity in this immigrant population [19], this lifestyle intervention addressing overweight Middle Eastern immigrants did not have the power to show significant correlations between glucose regulation (assessed as fasting glucose and insulin sensitivity) with Pro-NT.

Although non-significant, a trend towards reduced fat intake in the intervention compared to the control group was observed during follow-up. Since Pro-NT is stimulated by fat intake [20], our data thus indicates that other mechanisms than only fat intake are involved in the release of neurotensin during non-surgical weight loss. Obese individuals are reported to have elevated levels of bile acid production. Studies shows that oral intake of the bile acid “chenodyoxycholic acid” stimulates the secretion of GLP-1, neurotensin and glucagon amongst other plasma hormones [21]. Another study shows that morbidly obese patients undergoing RYGB have higher proneurotensin levels after 3 and 6 months compared to patients undergoing gastric banding although equal weight loss thus suggesting surgical procedures result in distinct alterations of gastrointestinal hormone metabolism. Thus, the possible mechanisms involve an interplay between gut hormones, bile acids and gut microbiota [22].

### Clinical implications

A growing proportion of the population is overweight and in Sweden today approximately 45% have a BMI > 25 kg/m^2^ [23]. Although neurotensin has been previously shown to predict cardiometabolic disease and cancer [10], in this RCT study of overweight individuals, we paradoxically observed increased release of neurotensin during non-surgical weight-loss. The observation is consistent with studies of mice and humans undergoing surgical weight-loss [16, 17]. Plasma Pro-NT rapidly increases after fat ingestion [24]. Dietary changes can affect peripheral Pro-NT function and body weight. One possible explanation is that peripheral Pro-NT in the intestine favors fat absorption and weight accumulation but central Pro- NT within the brain reduces feeding and increases physical activity behaviors that favor weight loss. Hence, it is possible that extremely elevated plasma Pro-NT levels after surgical and non-surgical weight loss procedures permit enough Pro-NT access to the brain to invoke centrally mediated behaviors that support weight loss [18, 25].

However, since weight-loss is demonstrated to reduce cardiovascular disease and mortality rate reduction [26–28], we conclude that the increased release of Pro-NT during weight loss may not contribute to increased cardiometabolic risk but rather reflect involvement of other mechanisms that promotes Pro-NT release other than dietary fats.

### Strengths and limitations of the study

The strength of this study is the randomised controlled design that reduces the risk of selection bias. Another strength is the thorough sampling of anthropometric, metabolic and lifestyle data. No harm to any participant was reported during the study.

A limitation was the participation rate; only one out of seven at high risk for type 2 diabetes agreed to participate in the study. Prominent causes of non-participation were related to problems taking time off work or finding childcare, reported by non-participants [11]. Other studies have reported higher participation rates when the intervention is proceeded by an educational intervention addressing knowledge barriers as well as the use of incentives [29]. Due to limited resources that was not feasible within this study.

Another limitation is the short study period. However, the ability of conducting long term lifestyle interventions in Middle Eastern immigrants is a challenge given the annual fasting month Ramadan impacting metabolism in both intervention as well as control groups. With a fairly low sample size, this study is rather a pilot study, and larger studies are needed to draw further conclusions of the role of neurotensin in weight loss.

## Conclusion

This RTC study of short duration shows that lifestyle induced weight loss was associated with increasing Pro-NT levels within the first four-months. Due to the previously published prognostic negative effects of high Pro-NT levels on morbidity and mortality, we conclude that long-term studies of change over time in Pro-NT following weight loss are needed.

## Data Availability

Data are available upon request as they contain potentially identifying information. Data access requests may be made to the data access group of the MEDIM study by contacting medim@med.lu.se.
